# Official promotion and extreme environmental regulation: Evidence from prefecture-level cities in China

**DOI:** 10.3389/fpubh.2022.1029869

**Published:** 2022-11-25

**Authors:** Zuhan Meng, Anna Shi, Sixuan Du

**Affiliations:** ^1^Business School, Hohai University, Nanjing, China; ^2^Yangtze River Protection and Green Development Research Institute, Hohai University, Nanjing, China

**Keywords:** official promotion, extreme environmental regulation, limited tenure, geographical rotation, systematic GMM model

## Abstract

Under the background of Chinese decentralization, avoiding the shortsightedness of local officials has become the policy focus of the central government's environmental regulation. In recent years, with the achievement of environmental protection gradually becoming a necessity for promotion, local officials increasingly prefer to implement extreme environmental regulation (EER) to quickly control environmental deterioration. Based on this specific fact, this paper constructs a systematic GMM model, trying to study whether the promotion of officials can fully explain the executive logic of EER under the influence of limited tenure and geographical rotation. It is found that EER and limited tenure have a U-shaped relationship, and geographical rotation can inhibit EER during the limited tenure. In addition, China has not formed an “environment-only” promotion tournament. Heterogeneity analysis showed that in areas with high bureaucratic compliance, local officials are late in strengthening EER during their tenure while the inhibition effect of geographical rotation is poor; Compared with older officials, young officials have stronger motivation to implement EER, and the inhibition of geographical rotation is obvious. To sum up, our research shows that the green promotion system only enhances officials' demands for environmental achievements, but it does not restrict officials' short-sighted governance strategies. The central government should start by expanding environmental protection assessment indicators and improving the quality of official rotation to narrow the implementation space of EER, thereby encouraging local governments to promote stable and sound environmental governance in a compliant and long-term manner.

## Introduction

China's economic growth miracle has won the world's attention, but the extensive development of only GDP has also been criticized. Huge environmental costs not only damage people's livelihoods and social welfare but also cause economic losses to China which account for 8–15% of GDP (1). Based on this, China has long been committed to improving environmental regulations to prevent and control pollution. However, China's unique decentralization system gives local officials extensive discretionary power. Under the environmental regulation system dominated by direct government control, local officials often choose the latter between the central policy orientation and their performance construction. Environmental regulation has a strong short-term and timing preference. Therefore, although the behavior of enterprises and the public will also impact the implementation effect of environmental policies in theory, the implementation deviation of China's environmental regulations is more directly related to the profit-seeking nature of local officials (2, 3).

Extreme environmental regulation (EER) is the product of this profit-seeking nature. Typically, Taiyuan City, Shanxi Province, China, entered the first batch of “winter clean heating pilot cities” in February 2017 on the policy signal of “developing clean heating in northern China.” The same year, in April, the Taiyuan Government took the lead in “pressurizing” all districts into “no coal areas” and cracked down on the storage, sale, and burning of coal in an all-round way. Then other prefecture-level cities in Shanxi Province also joined the follow-up brigade, which eventually led to many problems such as “no heating in cold winter”, “no normal life” and “no normal business”. In 2017, the secretary of Linfen Municipal Party Committee in Shanxi Province revealed in an interview: “Since November 2016, enterprises and construction sites in Linfen City have been forced to stop production. As of September this year, 600 enterprises have been violently banned and 384 enterprises have been suspended from rectification. The GDP has lost at least more than 3 billion yuan and the growth rate has slowed down by 2.8% points.” For a long time, the implementation of similar environmental policies has frequently appeared in different forms. For example, in 2009, many small and medium-sized enterprises are completely shut down under the steel production restriction order, and in 2013, the “two high” industries such as cement were cut off under the energy saving and emission reduction policy (4).

Compared with general environmental regulation, EER has two characteristics: First, unconventional. Local officials ensure that environmental pollution can be controlled in a short time by implementing unconventional measures (5); Second, undifferentiated. Regardless of whether local companies have violated regulations and regardless of the stage of the development of the local industrial structure, local officials uniformly implement undifferentiated “one-size-fits-all” measures. In the past decade or so, EER has occurred frequently (6). We cannot help but wonder why is this violent aesthetics, which dampens regional economic vitality with expensive environmental protection costs, repeatedly favored by local officials?

The academic community believes that there are two views on the universality of EER: First, EER is an expedient measure to complete the central task under the tight implementation time and the sudden rise of environmental protection pressure. Zhuang and Hu (7) point out that when governance resources cannot match the difficulty of governance, local officials prefer to adopt EER compared with centralized governance. Second, EER is a competitive strategy to gain promotion weight under the pressure of officials' assessment and the need for performance construction. The objectively existing competitive game between local governments have intensified the local officials' motivation to whitewash environmental performance. It happens that the immediate effect of EER is more likely to transmit political performance signals (8). Both viewpoints are self-consistent but different from each other in explaining the logic of local officials' implementation of EER. The former tends to officials passively responding to environmental protection tasks under environmental accountability, while the latter tends to officials actively demonstrating environmental performance under political incentives.

Whether it is “passive response” or “active demonstration”, this paper argues that the main goal of local officials is always to complete the assessment indicators within a limited tenure to qualify for promotion. In the early days, China put forward the famous assertion that “development is the last word.” The absolute link between economic growth and promotion qualifications motivated local officials to devote most of their resources to economic construction (9). At the same time, the soft constraint indicators of some environmental tasks were not valued (10), and local officials even formed interest alliances with local polluting enterprises to obtain stable financial revenue (11). Therefore, in this period, in order to win the promotion tournament, most local officials adopt the logic of “passive response” to implement EER.

However, the central government gradually realized that the environmental accountability system lacking promotion incentives has apparent principal-agent losses. In 2007, the environmental protection “one-vote veto” system was officially incorporated into the promotion assessment system. Local officials began to spontaneously improve the degree of management of the ecological environment indicators in the assessment system when formulating regional development strategies. In 2015, the “Opinions on Accelerating the Construction of Ecological Civilization” promulgated by the State Council made it clear that the leading group and leading cadres who have not fulfilled the energy conservation and emission reduction goals shall not be transferred to important positions or promoted. The general trend of determining rewards and punishments through ecological performance index ranking is gradually taking shape. Studies have confirmed that outstanding environmental achievements will indeed increase the promotion probability of officials (12–14), and the central government is using powerful administrative means to encourage local officials to fight pollution prevention and control battle. However, pollution control is a complex and continuous systematic project. Local officials affected by job mobility generally lack enthusiasm for establishing a long-term environmental governance mechanism of “making wedding dresses for others” (15). Therefore, during this period, in order to obtain promotion tickets, most local officials adopt the logic of “active demonstration” to implement EER, although this kind of short-term “ecological management” may be sub-healthy.

Compared with previous studies, the possible marginal contributions of this paper are as follows: First, it empirically tests the impact of official promotion on EER. Existing studies have confirmed the impact of official promotion on the effect of environmental regulation in terms of energy productivity (16), energy efficiency (13), and air quality (17). However, few studies have explored the dynamic changes of environmental regulation behavior itself driven by promotion. Based on the background of rising environmental priority (18, 19), this paper thoroughly explains the logic of officials implementing EER out of the motive of “active demonstration”, and provides suggestions for optimizing the promotion system for officials and motivating local officials to adopt reasonable regulation strategies in the future.

Second, it proves that there has not yet been an “environment-only” promotion tournament in China. The parallel effect and coordinated promotion of the environmental tournament and the GDP tournament have been formed (20). Some works of literature have proposed that the crowding effect of economic performance on environmental governance in the promotion assessment system gradually becomes a supporting effect. Based on this, this paper incorporates the indicators that symbolize economic growth, social welfare, and environmental protection investment into the regression, and the results show that the premise of the implementation of EER is still reasonable economic growth.

Third, the heterogeneity analysis is carried out from the aspects of bureaucratic compliance and official age. Since the strong bureaucratic compliance in the region may reduce the enforcement space of EER, and differences in the age of officials may also lead to officials adopting different regulatory strategies, this paper groups the samples according to the bureaucratic compliance and the age of officials. Empirical evidence shows that the timing and intensity of implementing EER within a limited tenure are indeed affected by bureaucratic compliance and the age of officials, and the moderating effect of geographical rotation also has sample heterogeneity. The heterogeneity analysis provides more comprehensive evidence for incentivizing local officials to build long-term and effective environmental governance mechanisms.

**Figure 1:**
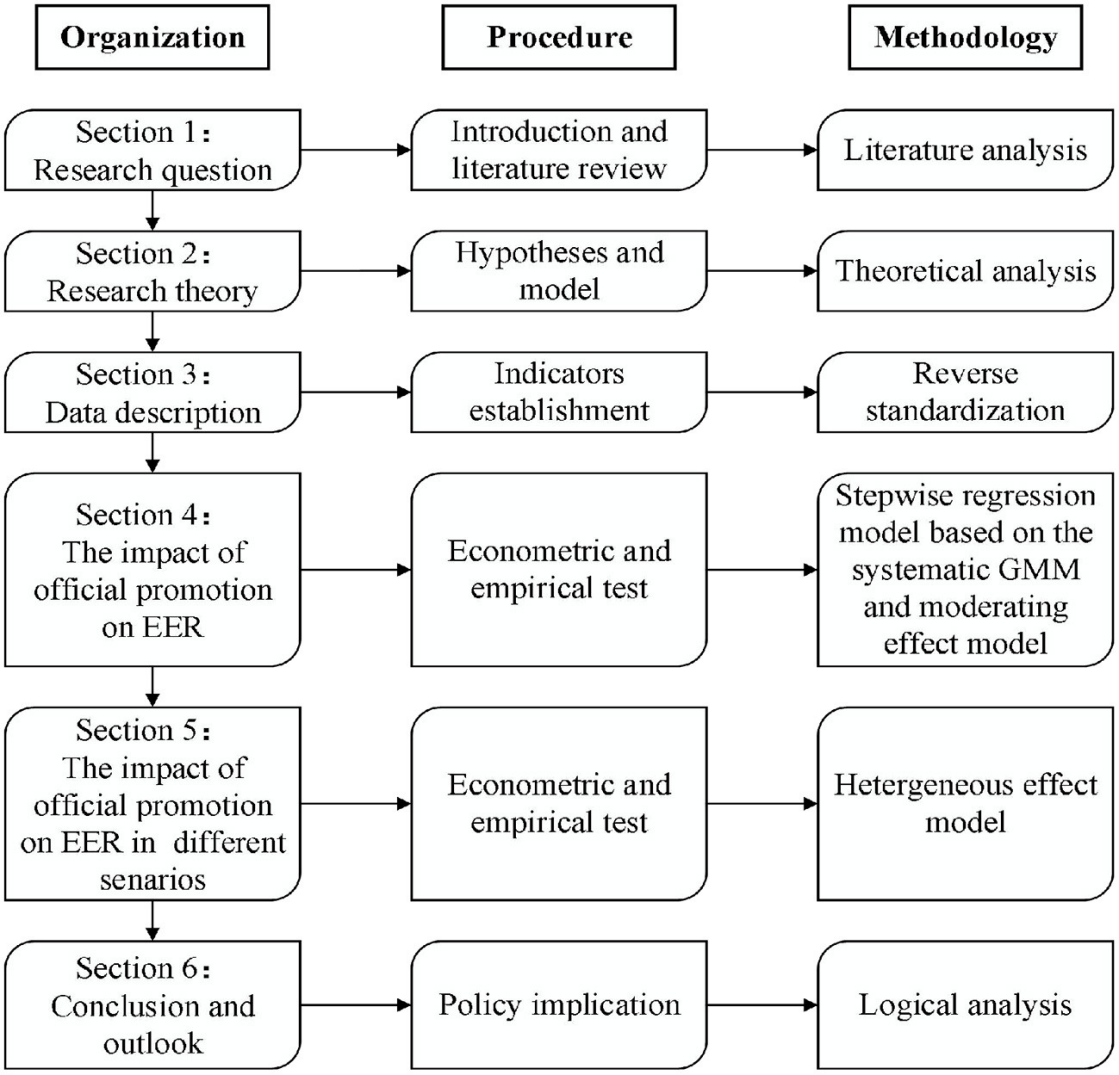
The logical framework of this paper.

The arrangement of this paper is as follows (see Figure 1 for the logical framework): The second part is research design; The third part is variable description and data sources; the fourth part is empirical results and analysis; the fifth part is further discussion; the sixth part is conclusion and outlook.

## Research design

### Theoretical analysis and research hypotheses

Under the condition of information asymmetry and insufficient supervision resources, the Chinese government tries to rely on the promotion and evaluation of officials to realize the transmission of the central will to the localities and the coupling of the interest structure (21, 22). At the beginning of the reform and opening up, the strong growth signal released by the central government formed an evaluation system for official promotion with GDP as the core. Therefore, starting with the “promotion tournament” hypothesis (23), many scholars attribute China's economic growth miracle to the official promotion system (24, 25). Political incentives have greatly intensified the competition of “chasing and competing with each other” among local governments. However, the economic growth model adopted for promotion purposes is not efficient (26). Problems such as high resource consumption, ecological damage, and environmental pollution have become inevitable products of single economic construction. As China's environmental situation became increasingly severe, the central government tried to sign “military orders” with local governments to ensure bottom-line environmental quality control. However, the result was that local officials lacking incentives adopted EER to avoid environmental protection accountability, which did not actually improve environmental indicators' management.

In recent years, in order to motivate local governments to set an objective function that prioritizes environmental protection, the central government has added binding environmental indicators to the official promotion assessment system, trying to guide local governments' competition direction from “competition for growth” to “competition for sustainability”. Zhang et al. (27) show that strict environmental indicators can strengthen the strategic interaction of the “race to the top” when local governments implement environmental policies. Wu and Cao (14) believe that after greening the professional incentive structure of local officials in China, local officials who are more “green” get more promotion opportunities.

Regrettably, the current assessment of green achievements by the central government may only enhance officials' manipulation of environmental indicators. Local government competition is the intermediate product of “competition for environmental protection achievements” in the transition from “competition for growth” to “competition for sustainability”. At this time, local officials will seek the maximum path of achievement construction in order to obtain promotion qualifications. Thus they cannot formulate a long-term environmental governance mechanism, which is in line with the choice of “rational people”. Zhang (28) proposes that the governance goals of local officials are only anchored in specific indicators rather than broad environmental protection of public goods. Yuan et al. (29) also believe that the setting of binding environmental protection indicators did not correct the lack of environmental protection awareness of local officials' short-sighted behavior. To sum up, although the central government expects to form an environmental tournament similar to “competing for growth” through “greening” official promotion system, EER of “competition for environmental achievements” may be more favored by local officials in fact.

Kamp (6) believes that local officials' enforcement of EER makes it easier for the central government to visually verify whether they are taking environmental protection actions. To put it simply, in the period of low promotion probability, the implementation of environmental regulations by local officials tends to “avoid responsibility”, that is, adopt a relatively flexible approach to environmental governance; In the period of high promotion probability, local environmental regulation implementation tends to “taking credit”, that is, maximizing the power and resources at hand in response to incentives from higher-level governments. In order to describe the influence of performance incentive level on officials' behavior and decision-making in different periods, the existing literature regards officials' tenure as an invisible governance mechanism between the central government and the local government to act as an agent for officials' promotion incentive (30–32). Specifically, Wu and Zhou directly regarded the tenure as the proxy variable of promotion incentives for officials, and examined the influence of promotion incentives for local officials on urban maintenance and construction (30). Cai et al. further shows that with the extension of tenure and improvement of administrative ability, officials need to make achievement as soon as possible in order to stand out in the “promotion tournament” (31). Zeng and Zhu hold the same view, and regard tenure as the endogenous factor of promotion incentive (32). Therefore, this paper attempts to discuss the influence of promotion incentives on EER in the political cycle of officials from two aspects: the limited tenure and the geographical rotation within the limited tenure.

Limited tenure is naturally associated with EER adopted by local officials. Although the limited tenure can actively defend local officials to form a network of local interests, it also creates a short-term preference for local officials to “seek for quick success” (33, 34). The nature of serving political interests will lead them to adopt EER to obtain visible political achievements. Existing studies have focused on economic growth performance (24, 35, 36), local fiscal expenditure bias (37), land approval (38), urban construction (39), etc, trying to explain the intrinsic link between limited tenure and the short-sighted behavior of local officials. Regarding the fluctuation characteristics of these observations in finite tenure, most scholars tend to support the view of a nonlinear relationship, that is, local officials have different decision-making preferences in different tenure stages.

Accordingly, we also interpret the logic of how limited tenure affects EER by different tenure stages: In the early days of taking office, rational local officials can judge that the excessive use of resources in environmental governance will crowd out other development in the jurisdiction resources, such as economic development, public safety, social security, etc. Most officials will not have obvious resource inclination in the field of environmental governance in the early days of tenure, and the tendency of EER is weakened; As the promotion node is approaching, the “last-ditch” mentality of local officials and the long-term accumulation of resources can give birth to immediate environmental performance projects (40, 41). Under the strong constraints of the one-vote veto system for environmental protection, EER can not only achieve the bottom line of environmental quality required by superiors to the greatest extent but also greatly simplify the steps required for emission reduction. It is expected that local officials who are eager to be promoted tend to strengthen EER in the later period of their tenure, and the construction of environmental performance shows an obvious “incentive effect of turnover”. So far, this paper proposes Hypothesis 1:

Hypothesis 1: EER exhibits U-shaped characteristics during officials' tenure.

Geographical rotation is an essential measure for China to promote coordinated regional development (42), and its excellent economic effects have been widely confirmed (43–45). However, the environmental effects of geographical rotation are still in their infancy in academia. Some scholars believe that geographical rotation can disintegrate the alliance of government-enterprise interests and promote regional cooperation while optimizing the effect of environmental governance. Liang and Gao (46) believe that geographical rotation can strengthen the enforcement of environmental supervision and reduce the illegal emission of pollutants by enterprises. Xiong et al. (47) hold the same point that frequent geographical rotation between local officials is conducive to the development of environmental protection in the inflowing regions. However, some scholars believe that geographical rotation may strengthen the short-sighted tendencies of local officials (42). In order to gain “political prestige” within a short rotation period, rotation officials take an efficient, low-quality approach to environmental regulation (48). Therefore, geographical rotation may also strengthen the extreme governance motives of local officials, which is not conducive to the construction of a long-term environmental governance mechanism. In summary, this paper proposes the following competing hypotheses:

Hypothesis 2a: Geographical rotation will inhibit the tendency of local officials to EER.
Hypothesis 2b: Geographical rotation will strengthen the tendency of local officials to EER.

### Research model and analytical logic

Based on the existing research, it can be inferred that local officials' environmental regulation is a dynamic game process with a strong time correlation, and the influence of past habits and preferences on current regulatory behavior cannot be easily ruled out. Therefore, this paper uses the dynamic panel model to verify the influence of officials' promotion incentives on EER. A prominent advantage of the dynamic panel data model is that it can overcome the problem of variable omission by controlling the fixed effect, and it can also overcome the problem of reverse causality. In the dynamic panel model, the Generalized Method of Moments (GMM) model is more suitable for panel data with the characteristics of “big N and small T”, and its estimation error decreases with the increase of N under given conditions. Especially compared with horizontal GMM and differential GMM, systematic GMM makes full use of the validity of samples and reduces the error degree of generalized moment estimator. Therefore, based on this paper's research object and data, we adopt the systematic GMM model as the benchmark model.

It is worth mentioning that an important application of systematic GMM model is to solve endogenous problems, especially weak instrumental variables, by effectively replacing endogenous variables with instrumental variables that are highly correlated with endogenous variables and have nothing to do with disturbance terms (49). However, other methods simply assume that the covariance between explanatory variables and error terms is zero, and that there is no heteroscedasticity. In this case, It is necessary to adopt instrumental variable method or some other quasi-natural experimental methods to correct the error of parameter estimates caused by endogeneity.

Whether or not the estimation of systematic GMM is valid needs to go through the tool variable over-recognition test and the sequence correlation test. Sargan test and Hansen test are the main methods for the over-recognition test of tool variables. Among them, Hansen test is robust to random disturbance terms and has a wider application range, so this paper uses Hansen test to verify the validity of tool variables. The original assumption of Hansen test is that instrumental variables are valid, so its statistics should not be significant. Specifically, the p-value should be greater than 0.1. At the same time, in order to prevent errors caused by too many tool variables, the p-value of Hansen test is the best in the range of (0.1, 0.25). The main method of sequence correlation test is Arrellano-Bond test. The random disturbance term after the first difference in systematic GMM can have first-order autocorrelation but cannot have second-order and higher-order autocorrelation. Therefore, this paper focuses on the results of AR (2) in Arrellano-Bond test.

Through the deduction of the relationship between official tenure and EER in the Theoretical Analysis and Research Hypotheses part, we think that EER may present a U-shaped feature during official tenure, so this paper includes the quadratic term of official tenure, and the benchmark model is as follows:


(1) $$\begin{array}{l} EER_{i,t}=\beta_{0}+\beta_{1}EER_{i,t-1}+{\beta_{2}Tenure}_{i,t}+{{\beta_{3}Tenure}_{i,t}}^{2}\\ \;+\alpha X_{i,t}+\mu_{i}+\nu_{t}+u_{i,t} \end{array}$$

Among them, i represents the prefecture-level city, t represents the year, and *X*_*i,t*_ represents the control variable, including *Age*_*i,t*_, *Gec*_*i,t*_, *Edu*_*i,t*_, *Fdi*_*i,t*_, *Dol*_*i,t*_, *Ep*_*i,t*_, and *Fis*_*i,t*_. μ_*i*_ and ν_*t*_ represent the regional effect and time effect, respectively. ε_*i,t*_ is random disturbance term. From the perspective of coefficients, β_1_ reflects that local officials' EER is influenced by previous environmental regulation strategies, which proves that the dynamic panel model is scientific and reasonable. β_2_ represents the linear relationship between officials' tenure and EER, β_3_ reflects the nonlinear characteristics of EER during officials' tenure. If β_3_ is greater than 0, it means that EER exhibits U-shaped characteristics during officials' tenure. If β_3_ is < 0, it means that EER exhibits inverted U-shaped characteristics during officials' tenure. The coefficient we focus on is β_3_.

Considering that the special geographical rotation system in China may break the continuity of tenure, this paper gradually adds geographical rotation, the interaction terms of official tenure and geographical rotation. The model is as follows.


(2) $$\begin{array}{l} \;EER_{i,t}=\beta_{0}+\beta_{1}EER_{i,t-1}+{\beta_{2}Tenure}_{i,t}\\ \;+{{\beta_{3}Tenure}_{i,t}}^{2}{+\beta }_{4}Rotation_{i,t}+\alpha X_{i,t}+\mu_{i}+\nu_{t}+u_{i,t} \end{array}$$

(3) $$\begin{array}{l} EER_{i,t}=\beta_{0}+\beta_{1}EER_{i,t-1}+{\beta_{2}Tenure}_{i,t}+{{\beta_{3}Tenure}_{i,t}}^{2}\\ \;{+\beta }_{4}Rotation_{i,t}+\beta_{5}\left(Tenure_{i,t}\times Rotation_{i,t}\right)+\alpha X_{i,t}\\ \;+\mu_{i}+\nu_{t}+u_{i,t} \end{array}$$

Among them, β_4_ indicates the net influence of geographical rotation on EER. β_5_ reflects the moderating effect of geographical rotation on the relationship between officials' tenure and EER. If β_5_ >0, it means that geographical rotation will strengthen the tendency of local officials to adopt EER; If β_5_ < 0, it means that geographical rotation will weaken the tendency of local officials to adopt EER. The coefficient we focus on is β_5_.

## Variable description and data sources

### Indicator description

#### Explained variables

Extreme environmental regulations (EER). Most of the previous studies refer to environmental regulation as local environmental protection revenue and expenditure or the reduction of pollutants (50, 51). However, EER is fundamentally different from traditional environmental regulation. First, EER is accompanied by high economic and social costs, so environmental protection revenue and expenditure alone cannot fully reflect the extreme degree of environmental regulation by local officials; Second, EER is characterized by implementing environmental policies in a convenient manner in a short period of time to build a good ecological performance, so the reduction of pollutants alone cannot reflect the political benefits brought to local officials by EER. Starting from bureaucratic compliance, Kamp (6) uses per capita income to measure EER, which means that when per capita income is low, local officials have less space and resources to fully implement environmental policies, so they are more likely to adopt EER. Since this paper attempts to study the relationship between official promotion and EER, there is an endogeneity problem between per capita income and official promotion, so this paper does not consider this measure.

This paper adopts the number of national key monitoring polluting enterprises to construct EER. Enterprises under national key monitoring, referred to as “national control point” enterprises, are polluting enterprises that are screened by local governments and actively reported to the central government based on the national environmental statistics database, and need to take the lead in implementing emission reduction responsibilities. After the report is completed, the local government is responsible for recording the pollution discharge situation of “national control point” enterprises month by month and publishing it regularly. As the official documents do not disclose the data about the distortion of environmental regulations in various regions from the front, we reverse the number of “national control point” enterprises reported by local governments as the proxy variable of EER. The logic of adopting the number of “national control point” enterprises in this paper lies in the fact that the number of “national control point” enterprises is not in the evaluation category of the current promotion appraisal system. Therefore, it can truly reflect the local officials' investment in building a long-term environmental governance mechanism and their attitude towards soft constraints on environmental protection while excluding the benefits of political achievements. In particular, the cumbersome process of reporting “national control point” enterprises, the differentiated monitoring of national control point enterprises, and pollutant discharge permit according to the national total amount control are in sharp contrast with the short-term, undifferentiated and unconventional nature of EER. Adopting the number of “national control point” enterprises also has the following advantages: (1) The annual data of this index will not be disturbed by the change of statistical standards of pollutants, and it has time continuity; (2) This indicator can reflect the environmental protection enthusiasm of local officials in real time, and its lag effect is smaller than that of emission reduction indicators. In particular, due to the rapid development of modern cities, automobile exhaust and coal for heating residents have caused serious air pollution. Compared with exhaust gas pollution, wastewater pollution can better represent the pollution generated by industrial enterprises. Therefore, we use the number of wastewater enterprises in “national control point” for index construction.

After manually matching the number of wastewater enterprises in “national control point” with 267 cities, we draw on the measurement method of industrial environmental regulation by Wang and Liu (52) to construct an index of EER. It consists of three steps: First, since local officials adopt EER and local self-reporting of “national control point” enterprises are two mutually exclusive environmental regulation strategies, this paper makes reverse standardization of “national control point” enterprises.


$$\begin{array}{l} pc_{i}^{s}=\frac{max\left(pc_{i}\right)-pc_{i}}{max\left(pc_{i}\right)-min\left(pc_{i}\right)} \end{array}$$

Among them, *pc*_*i*_ represents the original value of the number of “national control point” enterprises in city i, *min*(*pc*_*i*_) *and max*(*pc*_*i*_) represent the minimum and maximum values of *pc*_*i*_, $pc_{i}^{s}$ represents the standard value of the original value after reverse processing.

Second, considering the differences in industrial pollution and economic development levels in different cities, which will affect our comparison of the number of “national control point” enterprises reported by local officials, an adjustment coefficient for the inverse value is added in order to analyze EER of local-level cities on a horizontal line. The calculation method of the adjustment coefficient is as follows:


$$\begin{array}{l} A_{i}=\frac{p_{i}}{\sum_{i}p_{i}}/\frac{GDP_{i}}{\sum_{i}GDP_{i}} \end{array}$$

Among them, $p_{i}/\underset{i}{\sum }p_{i}$ is the proportion of the industrial wastewater discharged by city i to the domestic industrial wastewater discharge; $GDP_{i}/\underset{i}{\sum }GDP_{i}$ is the proportion of the gross product of city i to the gross domestic product. The logic of the adjustment is: if the pollution intensity of the city is higher, the same number of “national control point” enterprises means stronger EER, so a greater weight should be given to the reverse value.

Third, construct EER according to the reverse value and adjustment coefficient:


$$\begin{array}{l} EER_{i}=pc_{i}^{s}\times A_{i} \end{array}$$

#### Explanatory variables

Official tenure (Tenure). Official tenure has been used by many works of literature as a proxy variable for official promotion incentives (30–32, 39). In China, the tenure of local officials is 5 years and can be re-elected, but the actual average tenure is short, with frequent replacements. Tian et al. find that 76% of the municipal party committee secretaries and 84% of the mayors in a single prefecture-level city have tenures of no more than 5 years (53), so this paper selects the tenure of prefecture-level city mayors as the tenure of local officials. The measurement of tenure follows existing research conventions (43): When there is a phenomenon of “tenure alternation”, officials with longer tenures are considered as local officials of prefecture-level cities in that year.

#### Moderating variables

Geographical rotation (Rotation). Geographical rotation are generally considered to be able to effectively reduce the inertia of officials and improve environmental efficiency by shortening their tenures (54). Therefore, it is necessary to explore the moderating effect of geographical rotation on the relationship between official tenure and EER. This paper adopts the 01 variable to measure the geographical rotation of local officials. If the official was transferred from another city before taking office, it is assigned a value of 1, otherwise, it is assigned a value of 0.

#### Control variables

This paper selects control variables from two dimensions of official characteristics and regional development.

Official characteristics. (1) Official age (Age). Young officials usually have better career prospects and therefore opt for more aggressive environmental strategies (55). (2) Educational background (Edu). Generally speaking, The higher the degree of local officials, the more they can recognize the necessity of environmental remediation. This paper uses the assignment method to quantify Edu. If the official has a bachelor's degree, it will be assigned a value of 1, a master's degree will be assigned a value of 2, a doctoral graduate student will be assigned a value of 3, and the rest will be assigned a value of 0. (3) Government-enterprise collusion (Gec). Under the background of information asymmetry between the central and local governments, government-enterprise collusion is a major obstacle to current environmental regulation (56). This paper uses whether officials are local as a proxy variable for Gec.

Regional development. (1) Foreign investment support (Fdi): Generally speaking, the capital and management experience brought by the large inflow of foreign businessmen may promote the upgrading of environmental protection technology (57), but there is also a “pollution shelter” effect (58), which induces EER. In this paper, using the research of Wang et al. (59) and Dean et al. (60) for reference, Fdi is included in the regression as a control variable. Specifically, Fdi is measured by adjusting the total foreign investment data to RMB denominated by the exchange rate. (2) Degree of industrialization (Doi). Doi reflects the proportion of extensive economy in a region. When the industrial output value accounts for a high proportion of GDP, the environmental pollution in this area will be aggravated (61), thus inducing local officials to take radical environmental governance strategies. Therefore, this paper will also include Doi in the regression. This paper uses the proportion of the secondary industry to measure Doi. (3) Ecological pressure (Ep): The concentration of population and production activities usually aggravates regional environmental pollution (62), and local officials are more likely to adopt EER. This paper uses population density to measure Ep. (4) Fiscal surplus (Fis): The impact of Fis on EER is unknown. It is included in the regression to control the accompanying interference of local protectionism and pollution transfer. This paper uses the ratio of fiscal revenue to fiscal expenditure to measure Fis.

### Data sources and descriptive statistics

Since cities are one of the primary contributors to energy consumption and greenhouse gas emissions (63), based on panel data of 267 cities in China, this paper selects 2009–2017 as the research period to explore the relationship between official promotion and EER. The “national control point” enterprises data comes from the website of the Ministry of Ecology and Environment of the People's Republic of China. Official data comes from official resumes on official websites of various local governments and the Baidu Encyclopedia. After crawling and summarizing, they are manually matched with cities. The regional data comes from the China Urban Statistical Yearbook, and the exchange rate comes from the website of the National Bureau of Statistics. It is worth noting that the missing data in this paper accounts for a small proportion of the total sample size, and the systematic GMM model will automatically eliminate the missing sample data in regression. Therefore, this paper is based on the officially disclosed data to ensure the authenticity of the results. Table 1 presents the descriptive statistics of the main variables.

**Table 1:** Descriptive statistics.

**Variable**	**Unit**	**Obs**.	**Mean**	**Std.Dev**.	**Min**	**Max**
*EER_*i,t*_*	–	2,401	1.198	1.245	0	23.95
*Tenure_*i,t*_*	year	2,583	2.584	1.407	0.6	11.167
*Rotation_*i,t*_*	–	2,583	0.149	0.357	0	1
*Age_*i,t*_*	year	2,580	51.519	5.07	34.25	67.333
*Edu_*i,t*_*	–	2,583	1.991	0.767	0	3
*Gec_*i,t*_*	–	2,583	0.025	0.155	0	1
*Fdi_*i,t*_*	10^8^yuan	2,670	1.427	3.286	0	46.408
*Dol_*i,t*_*	%	2,668	48.412	9.965	13.85	82.24
*Ep_*i,t*_*	person/km^2^	2,655	453.802	336.395	4.97	2648.11
*Fis_*i,t*_*	%	2,670	2.667	1.578	0.649	18.025

## Empirical results and analysis

### Benchmark regression

In order to show as clearly as possible the introduction process of the core explanatory variables and their impact on the regression results, we present them in a step-by-step manner. All regression analyses adopt the robust standard error at prefecture level, and control the time fixed effect and regional fixed effect. This part examines the basic implementation of EER during the tenure of officials. The estimated results are shown in Table 2. All models passes the Hansen test and the first-order and second-order serial correlation tests, which proves that the instrumental variables in the model estimation process are properly selected, and the model results are not affected by the second-order serial correlation.

**Table 2:** Benchmark regression results.

**Variable**	**Equation(1)**	**Equation(2)**	**Equation(3)**
*EER_*i, t*−1_*	0.308^**^(2.20)	0.285^**^(1.95)	0.318^**^(2.06)
*Tenure_*i,t*_*	−0.995^**^(−2.14)	−0.992^**^(−1.97)	−1.007^**^(−2.21)
* ${\text{Tenure}}_{i,t}^{2}$ *	0.175^**^(2.21)	0.175^**^(2.04)	0.218^***^(2.72)
*Rotation_*i,t*_*		0.381(0.63)	5.100^**^(2.00)
*Rotation_*i,t*_*×*Tenure_*i,t*_*			−1.674^*^(−1.91)
*Age_*i,t*_*	0.051(1.62)	0.054(1.58)	0.051(1.40)
*Gec_*i,t*_*	−12.217(−1.49)	−14.14(−1.57)	−13.379(−1.38)
*Edu_*i,t*_*	0.120(0.78)	0.129(0.76)	0.149(0.85)
*Fdi_*i,t*_*	−1.588^**^(−2.11)	−1.680^**^(−1.99)	−1.741^**^(−2.37)
*Dol_*i,t*_*	−0.144^***^(−3.06)	−1.59^***^(−3.37)	−0.171^***^(−3.40)
*Ep_*i,t*_*	−0.008(−1.28)	−0.009(−1.41)	−0.008(−1.18)
*Fis_*i,t*_*	−1.709^***^(−2.95)	−1.886^***^(−3.35)	−1.907^***^(−3.23)
*Constant*	16.665^***^(2.69)	18.263^***^(3.15)	18.227^***^(2.85)
*μ_*i*_*	Yes	Yes	Yes
*ν_*t*_*	Yes	Yes	Yes
*AR(1)*	0.068	0.070	0.066
*AR(2)*	0.634	0.638	0.563
*Hansen test*	0.168	0.264	0.245
*Obs*	2070	2070	2070

T-value in parentheses;

* 10%,

** 5%, and

*** 1% significant levels, respectively.

From the regression results in column (1) of Table 2, it can be seen that the coefficient value of ${\mathrm{Tenure}}_{i,t}^{2}$ is greater than 0 and passes the 5% significance test (β_3_ = 0.175, *p* = 0.028), indicating that *Tenure*_*i,t*_ and *EER*_*i,t*_ have a U-shaped relationship. Its economic implication is that when officials take office at the beginning, their tendency to implement EER is weakening, but when they reach a certain tenure, their tendency to implement EER is constantly increasing, which shows that local officials in office do have the stage preference of implementing EER. After calculation, the inflection point of the U-shaped curve is located around 2.8 years after the official took office, which is within the data sample interval. It proves that the conclusion is not confused by the lack of sample support and is realistic. Local officials will strengthen the implementation EER around 2.8 years after taking office.

As far as the left side of the inflection point is concerned, the tendency of local officials to implement EER weakens with the increase of tenure. Newly appointed local officials are conducive to breaking the inherent “interpersonal network” in their jurisdictions, promoting information exchange between central and local governments, and reducing the negative impact of administrative inefficiency on local environmental governance. At the same time, local officials will not rashly set unattainable performance goals in the field of environmental governance, but under the environmental regulation characterized by “avoiding responsibility”, they are in the execution state of “not seeking merit, but not fault”. In China, the so-called “three fires when a new official takes office” means that the newly appointed officials will generally have a stronger motivation to plan the macro-strategy of the region in charge. With the central government's requirement for local government's performance appraisal changing from GDP-only theory in the past to diversified performance construction at present, local officials have to accomplish not only the economic task of innovation-driven transformation but also the livelihood task of improving the ecological environment and the political task of ensuring local social stability. In this case, the premature inclination of limited resources to a single field will hinder the comprehensive development of the jurisdiction. Therefore, local officials will not adopt “expensive” EER in the early days of taking office. The empirical results show that the initial negative relationship between official tenure and EER is in line with expectations. This is consistent with the results of Chen and Li (64) and Ma (40).

On the right side of the inflection point, after 2.8 years in office, local officials began to strengthen EER. The uniqueness of local officials' tenure in China lies in the flexibility of tenure (each tenure is not limited to 5 years). Under the non-fixed tenure system, local officials are no longer limited to adopting strategic environmental policies only in the year of change, but allocate target tasks according to factors such as tenure expectations and promotion nodes. The third year of an official's tenure is usually a period when their promotion opportunities are greatly improved (31, 65). Therefore, the 2.8-year time node is in line with reality, and EER has obvious “incentive effect of changing term”. At this time, local officials facing strong environmental protection constraints will first use the resources accumulated in the early tenure to ensure the bottom-line control of environmental quality, and then the mentality of rushing for promotion can give birth to unconventional means characterized by “taking credit” to highlight environmental protection achievements. EER, as an undifferentiated means characterized by “one size fits all”, can increase the “visible” environmental achievements of the higher-level government at the fastest speed in critical employment years. However, implementing this “cramming” policy also reflects the mobilization mode of China's local government with “promotion-driven” as the core and the regularization of sports governance. So far, the hypothesis has been verified, that is, EER has U-shaped characteristics in the tenure of officials.

From the regression results in column (3) of Table 2, it can be seen that with the gradual addition of the interaction term, the coefficient value of ${\mathrm{Tenure}}_{i,t}^{2}$ is still positive, and it has passed the 1% significance test (β_3_ = 0.218, *p* = 0.007). The influence degree is obviously greater than that in column (1). It can be seen that geographical rotation is helpful to further interpret the relationship between *Tenure*_*i,t*_ and *EER*_*i,t*_. When the main effect is still significant, the coefficient of *Rotation*_*i,t*_ × *Tenure*_*i,t*_ passes the 10% significance test and the coefficient value is less than 0 (β_5_ = −1.674, *p* = 0.057), indicating that geographical rotation can negatively moderate the relationship between EER and tenure. Its economic meaning is that geographical rotation can suppress EER during official tenure. The theoretical basis is that officials involved in geographical rotation can reduce the problem of “local protectionism” by disintegrating the interest alliance solidified by local officials for a long time, and force polluting enterprises to adopt cleaner production methods to promote the decoupling of economic output and pollution emissions (47). Stimulated by promotion, local officials tend to actively use their personal resources to strengthen exchanges and cooperation with their former places of employment, and influence their decision-making in their new places of employment. Specifically, officials in geographical rotation copy and extend the relevant decision-making experience of environmental governance, ecological protection and industrial transformation to the new employment areas, and guide the green technology innovation by promoting the investment attraction of the inflow places. Therefore, hypothesis 2a is supported.

As a accompanying result of the control variables, the control variables of official characteristics are not significant, while the control variables of regional development convey rich information. Among them, the coefficient of *Fdi*_*i,t*_ is significantly negative, and it passes the significance test of 5%, which means that in areas with more Fdi, local officials seldom implement EER. This may be because Fdi can alleviate the pressure of environmental pollution by improving the level of independent innovation, so as to reduce extreme governance. This conclusion is not only in line with the empirical expectation, but also consistent with the research of Dong et al. (66). The coefficients of *Dol*_*i,t*_and *Fis*_*i,t*_ are significantly negative, and they passes the significance test of 1%, which shows that there is less room for EER in areas with higher Doi and richer Fis. This is an interesting conclusion, and we think it may be related to bureaucratic compliance. This is discussed further in the next chapter.

### Robustness test

The previous analysis has drawn two basic conclusions: one is that there is a significant U-shaped relationship between official tenure and EER, and the other is that geographical rotation can inhibit extreme governance behaviors driven by promotion. This part further tests the robustness of the above conclusions in three aspects: replacing the explained variables, increasing the control variables and reducing the sample size. The results are shown in Table 3.

**Table 3:** Robustness test.

**Variable**	***PITI_*i,t*_***	***EER_*i,t*_***	***EER_*i,t*_***
	**(1)**	**(2)**	**(3)**
*PITI_*i, t*−1_*	0.453^***^(4.29)		
*EER_*i, t*−1_*		0.502^***^(4.35)	0.375^**^(2.49)
*Tenure_*i,t*_*	−0.313^**^(−2.07)	−0.669^*^(−1.75)	−0.859^**^(−1.95)
${\mathrm{Tenure}}_{i,t}^{2}$	0.041^*^(1.68)	0.164^**^(2.54)	0.194^**^(2.37)
*Rotation_*i,t*_*	0.510^**^(2.03)	9.052^**^(2.43)	5.235^**^(2.12)
*Rotation_*i,t*_*×*Tenure_*i,t*_*	−0.136^*^(−1.81)	−1.930^**^(−2.20)	−1.726^*^(−2.05)
*Control Variables*	Yes	Yes	Yes
*μ_*i*_*	Yes	Yes	Yes
*ν_*t*_*	Yes	Yes	Yes
*AR(1)*	0.000	0.079	0.059
*AR(2)*	0.652	0.917	0.711
*Hansen test*	0.130	0.519	0.145
*Obs*	915	1976	1882

T-value in parentheses;

* 10%,

** 5%, and

*** 1% significant levels, respectively.

#### Robustness test I: Other measures of EER

In the previous empirical analysis, the index construction of EER is obtained by the reverse processing of the number of polluting enterprises reported by the local government. However, it may be more appropriate to measure EER on the basis of the China Pollution Sources Regulatory Information Disclosure Index (PITI Index). The evaluation items of the PITI index mainly include 8 secondary indicators, such as “daily exceeding standards and violation records”, “enterprise environmental behavior evaluation”, “environmental supervision and petition and complaints”, etc., which can fully reflect the environmental governance attitude of local officials. Sadly, less than half of Chinese cities (113) were assessed by the index. In order to avoid the incompleteness of the data and increase the robustness of the conclusions, this paper uses the PITI index as a proxy index for EER in the robustness test after reverse processing. The PITI index comes from the official website of Institute of Public and Environmental Affairs (IPE).

From the regression results of column (1) in Table 3, it can be seen that the coefficients of ${\mathrm{Tenure}}_{i,t}^{2}$ and *Rotation*_*i,t*_ × *Tenure*_*i,t*_ are still robust (β_3_ = 0.041, *p* = 0.097; β_5_ = −0.136, *p* = 0.073), but the significance is lower than that of the benchmark regression results. Between them, the coefficient of *Rotation*_*i,t*_ × *Tenure*_*i,t*_ has increased from −1.674 to −0.136. Its economic meaning is that the inhibition of geographical rotation on EER during the officials' tenure has been reduced. This may be because the prefecture-level cities that voluntarily participate in the disclosure of the PITI index have a more standardized environmental regulation mechanism, which limits the space for EER by local officials, so the inhibitory effect of geographical rotation is not obvious.

#### Robustness test II: Control variables and others

The benchmark regression results show that in the critical period of promotion, local governments will strengthen EER, but considering the high economic and social costs and limited jurisdictional resources that are accompanied by EER, this paper further adds three indicators to describe economic growth, social welfare and environmental protection expenditure on the benchmark regression model, trying to test whether China's EER is constrained by economic or other social welfare indicators while validating the benchmark regression results. Among them, economic growth is measured by per capita GDP, social welfare is measured by the unemployment rate, and environmental protection expenditure is calculated by multiplying the ratio of local fiscal expenditure and provincial environmental protection expenditure to fiscal expenditure. The data comes from the China Urban Statistical Yearbook and the National Bureau of Statistics.

The regression results in column (2) of Table 3 show that the coefficient and significance of the original variables have not changed significantly (β_3_ = 0.164, *p* = 0.012; β_5_ = −1.930, *p* = 0.028), and the original conclusion is robust. Among them, the coefficient of per capita GDP growth rate is significantly positive, and the coefficients of the unemployment rate and local environmental protection expenditure are not significant, which means that when local officials adopt EER, they still need to take reasonable growth of the local economy as the premise. This is consistent with Kamp's (6) conclusion. Consistently, he suggests that high levels of economic growth may further drive blunt regulation. Therefore, although the priority of environmental indicators in the current promotion assessment system has increased, resulting in the increased overall management of environmental indicators in local behavioral decision-making, China has not yet formed an “environment-only” promotion tournament, which also proves views of Hu and Zong (20).

#### Robustness test III: Remove provincial capitals

Generally speaking, the law enforcement compliance of provincial capital cities is much higher than that of non-provincial capital cities. In order to prevent the bias of sample selection, this paper removes the sample of provincial capital cities and regresses. The results obtained are shown in column (3) of Table 3.

It is showed that after removing the sample of provincial capital cities, the U-shaped relationship between official tenure and EER and the inhibitory effect of geographical rotation still hold significantly (β_3_ = 0.194, *p* = 0.019; β_5_ = −1.726, *p* = 0.041). So far, three robustness tests have been passed, and the conclusions of this benchmark regression are reliable.

## Further discussion

### Bureaucratic compliance heterogeneity

Because *Dol*_*i,t*_ and *Fis*_*i,t*_ have a significant negative correlation with *EER*, this paper argues that regions with higher Doi and greater Fis may have stronger bureaucratic compliance, the enforcement space for EER is relatively smaller. Therefore, we attempt to perform a group regression on the sample from the perspective of bureaucratic compliance.

First, since the eastern region of China has a higher level of economic development and stronger administrative management capabilities than the central and western region, the corresponding bureaucratic compliance is also higher. Therefore, this paper divides the urban sample into the eastern region and the central and western region for heterogeneity regression.

Table 4 shows that the empirical results of the eastern and the central and western region are still consistent with the benchmark regression results. In terms of the U-shaped relationship between official tenure and EER, the inflection point in the eastern region is 0.2 years later than that in the central and western region, and the U-shaped curve is flatter, which means that officials in regions with stronger compliance may be subject to more stringent regulations. Due to the strong management constraints, the time to strengthen EER within a limited tenure is postponed, and the intensity of EER is weaker. In terms of the negative moderating effect of geographical rotation, the negative moderating effect in the central and western regions (β_5_ = −1.679, *p* = 0.000) is more obvious than that in the eastern regions (β_5_ = −0.184, *p* = 0.055), which means that in regions with weak compliance, geographical rotation is more likely to break the “alliance of interests” formed by local officials for a long time in power, and suppress extreme governance behaviors driven by promotion.

**Table 4:** Empirical results of official compliance heterogeneity I.

**Variable**	**Central and Western**		**Eastern**	
*EER_*i, t*−1_*	0.516^***^(6.91)	0.494*(5.67)	0.671^***^(5.56)	0.701^***^(6.23)
*Tenure_*i,t*_*	−0.912^**^(−2.12)	−0.793*(−1.71)	−0.460^**^(−2.31)	−0.484^**^(−2.11)
Tenure${\mathrm{Tenure}}_{i,t}^{2}$	0.157^**^(2.16)	0.141^**^(2.00)	0.075^**^(2.23)	0.084^**^(2.18)
*Rotation_*i,t*_*		6.653^***^(3.77)		0.925^*^(1.73)
*Rotation_*i,t*_*×*Tenure_*i,t*_*		−1.679^***^(−3.61)		−0.184^*^(−1.94)
*Control Variables*	Yes	Yes	Yes	Yes
*μ_*i*_*	Yes	Yes	Yes	Yes
*ν_*t*_*	Yes	Yes	Yes	Yes
*AR(1)*	0.013	0.002	0.076	0.078
*AR(2)*	0.223	0.242	0.341	0.331
*Hansen test*	0.316	0.206	0.526	0.484
*Obs*	1308	1308	762	762

T-value in parentheses;

* 10%,

** 5%, and

*** 1% significant levels, respectively.

Secondly, in view of the frequent adjustment of administrative divisions in many prefecture-level cities in China, this paper also starts from the urbanization rate and divides the sample of prefecture-level cities across the country into two groups according to the median (67). High urbanization rates mean higher bureaucratic compliance. The urbanization rate is measured by the resident population of prefecture-level cities in the total population of the jurisdiction. The regression results in Table 5 show that compared with areas with lower urbanization rate, the U-shaped inflection point in areas with high urbanization rate is delayed by 0.5 years, and the U-shaped curve is flatter, but the negative moderating effect of remote communication is not significant enough. The conclusions are basically consistent with Table 4.

**Table 5:** Empirical results of official compliance heterogeneity II.

**Variable**	**Low Urbanization**		**High Urbanization**	
*EER_*i, t*−1_*	0.583^***^(9.07)	0.593^***^(8.35)	0.620^***^(8.96)	0.648^***^(9.72)
*Tenure_*i,t*_*	−0.258^**^(−1.98)	−0.205^*^(−1.76)	−0.156^**^(−2.03)	−0.212^**^(−1.71)
${\mathrm{Tenure}}_{i,t}^{2}$	0.047^**^(2.11)	0.040^**^(2.06)	0.024^**^(2.06)	0.041^***^(2.66)
*Rotation_*i,t*_*		3.614^***^(3.01)		0.915*(1.74)
*Rotation_*i,t*_*×*Tenure_*i,t*_*		−0.684^***^(−2.88)		−0.350^*^(−1.77)
*Control Variables*	Yes	Yes	Yes	Yes
*μ_*i*_*	Yes	Yes	Yes	Yes
*ν_*t*_*	Yes	Yes	Yes	Yes
*AR(1)*	0.063	0.039	0.073	0.048
*AR(2)*	0.815	0.494	0.302	0.218
*Hansen test*	0.470	0.547	0.217	0.245
*Obs*	866	866	851	851

T-value in parentheses;

* 10%,

** 5%, and

*** 1% significant levels, respectively.

### Official age heterogeneity

An important reason for the failure to find a significant effect of official age in the benchmark regression is that the true effect of official age may be nonlinear. Generally speaking, municipal party secretaries and mayors at the bureau level have a high probability of facing the end of their political careers after the age of 54–55, so there is a clear downward jump in promotion incentives (62, 68). Based on this important observation, this paper argues that for officials of different ages, the relationship between official tenure and EER may exhibit different characteristics. In order to examine this heterogeneity, this paper conducts a subsample regression on young officials and old officials. Among them, officials younger than 55 years old are young officials, and officials older than 55 years old are old officials.

The estimation results of the sample of young officials in Table 6 show that EER still exhibits a significant U-shaped relationship within a limited tenure (β_3_ = 0.207, *p* = 0.023). Comparing the estimated results in column (1) of Table 2, after excluding the sample of older officials, the inflection point of the U-shaped relationship is slightly advanced, and the U-shaped curve is steeper, which indicates that young officials have more room for promotion and have stronger incentives for EER over longer tenures. The estimation results of the sample of old officials in Table 6 show that there is no significant correlation between EER and tenure after excluding the sample of young officials, but the coefficient of *Age* has a significant negative correlation with *EER*. For senior officials over the age of 55, promotion is difficult to motivate them to build ecological engineering achievements at the expense of high economic and social costs, and promotion incentives continue to weaken as retirement approaches. The failure to find a significant effect of official age in the benchmark regression stems from the bias of the younger sample. In addition, the negative moderating effect of geographical rotation is only significant in the sample of young officials, indicating that the central government can suppress the extreme governance motives of young officials through geographical rotation.

**Table 6:** Empirical results of official age heterogeneity.

**Variable**	**Young officials**		**Old officials**	
*EER_*i, t*−1_*	0.257^**^g(2.13)	0.323^***^(2.71)	0.227(0.84)	0.118(0.86)
*Tenure_*i,t*_*	−1.142^**^(−2.28)	−0.858^**^(−2.22)	−0.647(−0.68)	−0.255(−0.71)
${\mathrm{Tenure}}_{i,t}^{2}$	0.207^**^(2.31)	0.185^***^(2.69)	0.090(0.54)	0.027(0.41)
*Rotation_*i,t*_*		5.094^**^(2.06)		0.295(0.42)
*Rotation_*i,t*_*×*Tenure_*i,t*_*		−1.424^**^(−2.28)		−0.077(−0.31)
*Age_*i,t*_*	0.199(1.6)	0.060(0.40)	−0.029^*^(−1.72)	−0.017^*^(−1.68)
*Control Variables*	Yes	Yes	Yes	Yes
*μ_*i*_*	Yes	Yes	Yes	Yes
*ν_*t*_*	Yes	Yes	Yes	Yes
*AR(1)*	0.066	0.058	0.083	0.081
*AR(2)*	0.787	0.870	0.391	0.453
*Hansen test*	0.156	0.179	0.284	0.552
*Obs*	1583	1583	324	324

T-value in parentheses;

* 10%,

** 5%, and

*** 1% significant levels, respectively.

## Conclusion and outlook

In the context of the central government consciously increasing the weight of environmental indicators in promotion assessment, this paper is based on the typical fact that local governments have adopted EER, taking the panel data of 267 prefecture-level cities from 2009 to 2017 as a sample, using systematic GMM model to examine the relationship between official promotion and EER from two aspects of limited tenure and geographical rotation, then further differentiates bureaucratic compliance and official age for heterogeneity regression. The study find that EER is driven by promotion and has a U-shaped characteristic in the limited tenure of local officials. Geographical rotation can inhibit extreme governance behavior driven by promotion. The implementation of EER by local officials requires reasonable economic growth as a prerequisite. In areas with strong bureaucratic compliance, local officials are late to strengthen EER, and the inhibitory effect of geographical rotation is poor; Affected by age, younger officials are more motivated to implement EER than older officials and the inhibitory effect of geographical rotation is obvious. The research conclusion of this paper is of great significance for optimizing the evaluation system of official promotion and building a long-term environmental governance mechanism. However, there are still some shortcomings in this paper. Firstly, due to the availability of data, the time span of this paper is from 2009 to 2017, which fails to show the interesting relationship between official promotion and EER in recent years; Secondly, based on the further test of benchmark regression, this paper finds that EER still needs the premise of reasonable regional economic growth. However, under the current promotion appraisal system, the change of priority order of economic and ecological indicators has not been further discussed. Thirdly, this paper finds that the exchange of officials in different places is conducive to restraining EER, but the heterogeneous influence of geographical rotation has not been verified. Based on this, in future research, we think that the above shortcomings can be solved by constructing the index system of EER, constructing the empirical model of economic and ecological indicators and the promotion probability of local officials, and dividing officials' geographical rotation into horizontal communication between local governments and vertical communication between central and local governments. Based on the existing conclusions of this paper, this paper puts forward the following suggestions:

First, appropriately extend the term of office of officials, and decompose local environmental protection indicators by multiple factors in the promotion appraisal system. The unique limited tenure system in China is an important reason for the short-sighted behavior of local officials. Due to their short tenure and strong demand for political achievements, local officials lack enthusiasm for constructing a long-term governance mechanism of “making wedding dresses for others”. In addition, EER is often accompanied by unconventional one-size-fits-all measures, and the high economic and social costs also determine that EER can only be used as a “cramming” behavior before the threshold of promotion, but not as a continuous policy output mode. Therefore, we believe that appropriately extending the tenure can guide local officials to establish a long-term environmental governance mechanism. The emergence of “political blue sky” means that green performance incentives can only elevate environmental governance within a limited tenure to a speculative level, rather than truly mobilize local officials' enthusiasm for environmental protection. EER is still constrained by the local economic base, which also proves the “weak incentive” effect of ecology itself on local officials. Therefore, this paper thinks that the promotion appraisal system can be optimized, and local environmental protection indicators can be decomposed from many factors, such as the efficiency of solving environmental disputes, the results of regional environmental governance cooperation, the participation of enterprises in environmental responsibility (69) and the results of public or third-party environmental assessment, instead of just including some ecological performance indicators or boundary values of environmental pollutants into the promotion appraisal system, so as to avoid the tournament system with index appraisal as the main factor distorting officials' environmental governance and exporting short-term policy results. In addition, before making environmental governance decisions, local governments had better make an assessment in advance from three aspects: economic feasibility, environmental friendliness and social fairness (70), so as to make sufficient preparations for realizing the coordinated environmental governance of central and local governments.

Second, properly expand the proportion of local officials' geographical rotation, especially the proportion of rotation from backward areas to developed areas. On the one hand, geographical rotation can broaden the consistent ruling concepts of local officials, and balance the relationship between economic development and environmental protection through more innovative and comprehensive consideration of issues. On the other hand, geographical rotation can also break the “alliance of interests” caused by the long-term rule of local governments, forcing the officials in charge to change their development thinking and reducing problems such as “local protectionism” and “collaboration between government and enterprises”. Therefore, after optimizing the remote exchange system, the efficiency of information transmission and policy coordination may be higher. In future research, this paper thinks that it is possible to further divide the rotation of officials from different places into horizontal rotation between local governments and vertical rotation between central and local governments, to verify the influence of heterogeneity on local environmental regulation.

Third, adjust and reform the governance system of local officials of different age groups. On the one hand, while adhering to the standard of “younger and better educated” officials selection, the government should consciously put an end to the self-interest behavior and short-sighted tendency of young officials, and control them to make great efforts to build performance projects when they face the term or age threshold. On the other hand, promotion may only motivate some young officials who have a strong desire for promotion and have sufficient execution time and resources, while for those old officials who have fewer opportunities for promotion and lower desires, they should give full play to their “experience transfer” role, making it a “guide” for young officials to grow up.

## Data availability statement

The datasets generated for this study are available on request to the corresponding author.

## Author contributions

ZM and AS: conceptualization and supervision. ZM and SD: data curation and methodology. ZM: formal analysis and investigation. ZM, AS, and SD: writing—review and validation. All authors contributed to the article and approved the submitted version.

## Funding

This work was supported by the National Social Science Fund of China (Grant No. 19AGL023). National Social Science Fund of China is China's main channel for supporting basic research in the field of scientific research, and it faces the whole country, focusing on subsidizing researchers in universities and scientific research institutions with good research conditions and research strength.

## Conflict of interest

The authors declare that the research was conducted in the absence of any commercial or financial relationships that could be construed as a potential conflict of interest.

## Publisher's note

All claims expressed in this article are solely those of the authors and do not necessarily represent those of their affiliated organizations, or those of the publisher, the editors and the reviewers. Any product that may be evaluated in this article, or claim that may be made by its manufacturer, is not guaranteed or endorsed by the publisher.

## Abbreviations

EER: Extreme environmental regulation
Tenure: Official tenure
Rotation: Geographical rotation
Age: Official age
Edu: Educational background
Gec: Government-enterprise collusion
Fdi: Foreign investment support
Dol: Degree of industrialization
Ep: Ecological pressure
Fis: Fiscal surplus.
